# Supplementary material for: A novel synthetic strategy for styrene–butadiene–styrene tri-block copolymer with high cis-1,4 units via changing catalytic active centres
Source: R Soc Open Sci. 2019 Jun 19;6(6):190536. doi: 10.1098/rsos.190536 (PMC6599772; doi:10.1098/rsos.190536)
Supplement: The effect of [PPh3]/[Ni] in the synthesis of SBS via changing catalytic active centers [file rsos190536supp1.docx]

**Supporting Information**

**A novel synthetic strategy for styrene-butadiene-styrene tri-block copolymer with high *cis*-1,4 units** **via changing** **catalytic active centers**

Jie Liu^1,2^, Xin Min^1^, Xuan Zhang^1^, Xiuzhong Zhu^1^, Zichao Wang^1^, Tong Wang^1^, Xiaodong Fan^1,^*

^1^Ministry of Education and Shaanxi Key Laboratory of Macromolecular Science and Technology, School of Science, Northwestern Polytechnical University, Xi’an, Shaanxi, 710129, China.

^2^School of materials scienece and Engineering, Shaanxi Province Key Laboratory of Catalytic Foundation and Application, Shaanxi University of Technology, Hanzhong, Shaanxi, 723001, China.

* Corresponding authors: xfand@nwpu.edu.cn

Table of Contents

1. The effect of [PPh_3_]/[Ni]……..…….…….…….……..…….…….……..……. . . S1
2. ^1^H NMR spectra of SBSs obtained with different [PPh_3_]/[Ni] ratio.………… S2

**Table S.1**. The effect of [PPh_3_]/[Ni] in the synthesis of SBS via changing catalytic active centers ^a^.

| Entry | [PPh_3_]/[Ni] | conv^b^  % | St cont^c^  % | *M*_n_^d^×10^4^  g/mol | *M*_w_/*M*_n_^d^ | *Cis*-1,4^e^  % |
| --- | --- | --- | --- | --- | --- | --- |
| 1 | 0 | 0 | 17.9 | 4.3 | 1.35 | 97.2 |
| 2 | 0.5 | 53.2 | 28.8 | 5.3 | 1.55 | 97.2 |
| 3 | 1 | 91.1 | 35.1 | 6.1 | 1.53 | 97.2 |
| 4 | 1.5 | 29.5 | 24.5 | 4.8 | 1.58 | 97.2 |
| 5 | 2 | 10.8 | 20.2 | 4.5 | 1.61 | 97.2 |

a. First step, [Li] = 0.05 mmol, [St] = 4 mmol. Second step, [B]/[Li]=1, [Ni] = 0.01 mmol, [Bd] = 6 mmol. Third step, [PPh_3_]/[Ni]=1, [St] = 2 mmol. b. conversion of monomer St in the third step. c. Detercmined by ^1^H NMR spectroscopy. d. Detercmined by SEC-MALLS. e. Measured by ^1^H NMR and ^13^C NMR spectroscopy.

As shown in Table S.1, the ratio of [PPh_3_]/[Ni] seriously affected the conversion of styrene during the third polymerization step of SBSs. The system displayed almost no catalytic activity towards styrene ([PPh_3_]/[Ni] = 0) after completion of the butadiene polymerization. When a small amount of triphenylphosphine ( [PPh_3_]/[Ni] = 0.5) was added to this system before the polymerization of styrene, the catalytic activity of the system to styrene was significantly improved, the conversion of styrene was more than 50%, and the catalytic activity to styrene of the system was highest when the the ratio of [PPh_3_]/[Ni] reached to 1. It was attributed to the fact that the electron density of catalytic active centers could be adjusted by adding a certain amount of electron-donating triphenylphosphine (PPh_3_). If the ratio [PPh_3_]/[Ni] was high than 1.5, the conversion rate of styrene would be gradually decreased, because an excess of triphenylphosphine could seriously reduce the coordination ability of the catalyst system.


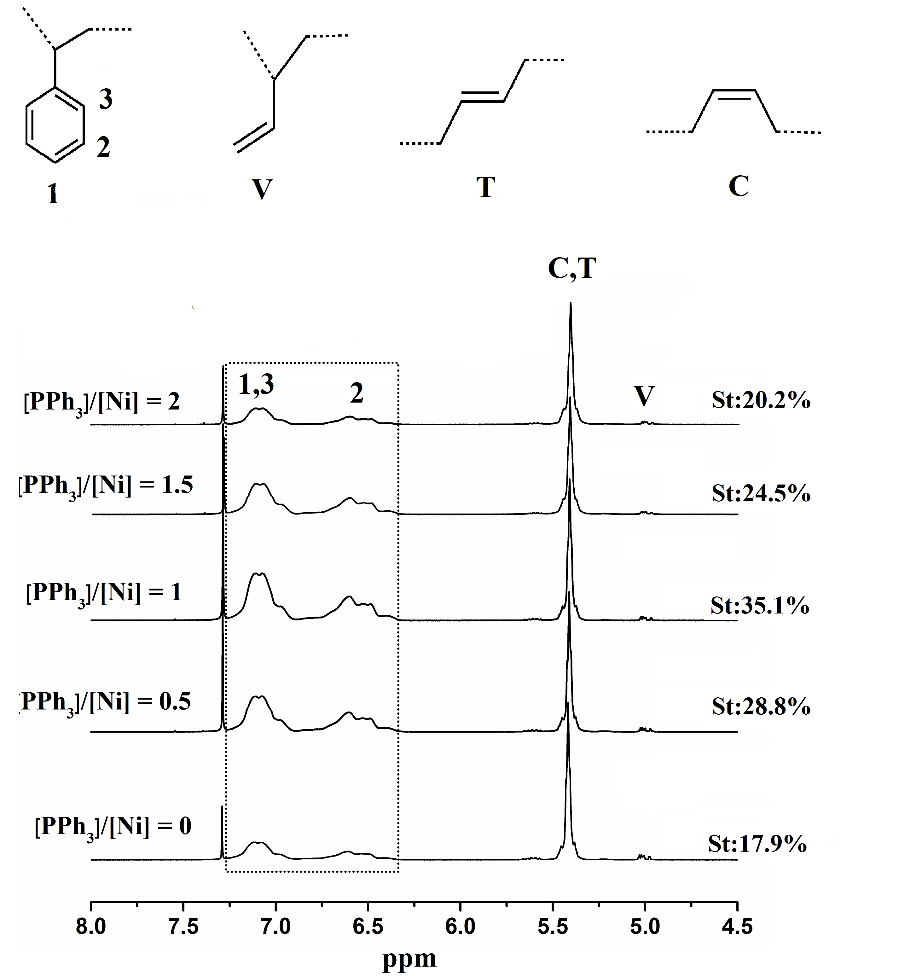


**Fig. S.2** ^1^H NMR spectra of SBSs obtained with different [PPh_3_]/[Ni] ratio.

As shown in **Fig S.2,** when the amount of triphenylphosphine [PPh_3_]/[Ni] = 1, the catalytic activity of the catalyst system towards styrene was the highest, which allowed a content of styrene in the third polymerization step to reach to 35.1%. According to the data of this ^1^H NMR and **Table S.1**, it could be calculated that the conversion rate of styrene in the third polymerization step was higher than 90%.
